# Assessing the efficacy and safety of Yinqiao powder-maxing Ganshi decoction in the treatment of the major symptoms of mild and moderate COVID-19 by telemedicine–study protocol for a randomized, double-blind, placebo-controlled trial

**DOI:** 10.3389/fphar.2023.1261338

**Published:** 2024-01-08

**Authors:** Chi Him Sum, Tong Wendy Li, Hongwei Zhang, Hing Yu Hung, Ben Yuk Fai Fong, Wai Ling Lin, Tak Yee Chow, Ka Chun Leung, Cho Wing Lo, Chon Pin Chia, Kam Leung Chan, Zhi-xiu Lin

**Affiliations:** ^1^ Hong Kong Institute of Integrative Medicine, The Chinese University of Hong Kong, Hong Kong, Hong Kong SAR, China; ^2^ School of Chinese Medicine, Faculty of Medicine, The Chinese University of Hong Kong, Hong Kong, Hong Kong SAR, China; ^3^ College of Professional and Continuing Education, The Hong Kong Polytechnic University, Hong Kong, Hong Kong SAR, China

**Keywords:** COVID-19, Chinese medicine, Yinqiao Powder, Maxing Ganshi decoction, randomized control trial, clinical trial

## Abstract

**Background:** The Coronavirus disease 2019 (COVID-19) is the largest global epidemic in recent time. Chinese medicine has been recognized by the World Health Organization as an effective treatment for COVID-19, but there is still a lack of high-quality randomized, double-blind trials using placebo as the control to support its application, which may hinder its further promotion locally and internationally.

**Objectives:** This study will evaluate the efficacy and safety of Yinqiao Powder-Maxing Ganshi Decoction with variation in relieving major symptoms of mild and moderate COVID-19 by telemedicine.

**Methods and design:** This clinical study is a randomized, double-blind, placebo-controlled trial that applies telemedicine to evaluate the efficacy and safety of Yinqiao Powder-Maxing Ganshi Decoction in the treatment of mild and moderate COVID-19. Eligible subjects will be randomly divided into either treatment or placebo groups for up to 14 days after stratification according to age (A:18–49, B:50–65) and the number of vaccinations (a: ≥3 doses, b: ≤2 doses). The treatment group will receive Yinqiao Powder-Maxing Ganshi Decoction granules along with certain variation based on their symptoms, and the placebo group will receive the same amount of placebo granules. Subjects will be prescribed different additions based on their symptoms and pathogenesis at the inclusion. The oral temperature, oximeter, result of rapid antigen test and symptom score will be recorded by subjects until they have stopped the medication. Subjects are required to have follow-up assessment by video-conference on days 7, 14 and 35. The time for the body temperature returning to normal will be used as the primary outcome.

**Discussion:** This trial will provide scientific evidence on the use of Yinqiao Powder-Maxing Ganshi Decoction for the treatment of COVID-19, and the results would help raise the awareness in Hong Kong and the international community on the use of Chinese herbal medicine for treating COVID-19.

**Clinical Trial Registration:** clinicaltrials.gov, identifier NCT05787327.

## Introduction

Coronavirus Disease 2019 (COVID-19), caused by the severe acute respiratory syndrome coronavirus 2 (SARS-CoV-2), has spread rapidly and is the largest worldwide epidemic in recent time. As at 25 June 2023, there were more than 767 million confirmed cases of COVID-19 globally, including more than 6.9 million deaths (World Health Organization, 2023).

Currently, western medicine including corticosteroids, antiviral drugs, immunomodulating agents, convalescent plasma therapy, anticoagulants and anti-inflammatory drugs are commonly used in the treatment of COVID-19 (Cascella et al., 2023). In Hong Kong, COVID-19 oral drugs (Paxlovid or Molnupiravir) would generally be prescribed in a hospital or clinic if the patient has no relevant contraindications and fulfills the clinical guidelines (COVID-19 patients age 60 years or older, high-risk groups, and people under age 60 years with chronic diseases) when the patient is diagnosed with COVID-19 and the onset of symptoms is within 5 days of presentation (Hospital Authority, 2023). Chinese medicine (CM) has a long history of preventing and curing disease and has played an important role in prevention and treatment of many epidemics. World Health Organization (WHO) launched a meeting on the use of CM in the treatment of COVID-19 in March 2022 (World Health Organization, 2022). It recognized the contribution of CM and encouraged adding CM to the existing treatment regimen for COVID-19. At the same time, the meeting also advocated to promote more international trials to further evaluate the effectiveness and safety of CM for COVID-19.

Currently, various CM interventional measures are available for COVID-19 treatment, of which the most famous are the “3 medicines and 3 formulations (三藥三方)”. “3 medicines (三藥)” mean 3 proprietary Chinese medicines, including Jinhua Qinggan Granule, Lianhua Qingwen Granule/Capsule and Xuebijing Injection Fluid; “3 formulations (三方)” are 3 herbal prescriptions, including Qingfei Paidu Decoction, Huashi Baidu Formula and Xuanfei Baidu Formula. The efficacy, safety and possible mechanism of “3 medicines and 3 formulations” have been supported and elucidated by a number of studies either used alone or in combination with usual care (Wang et al., 2020b; Cheng and Li, 2020; Duan et al., 2020; Liao et al., 2020; Zhao et al., 2020; Sun et al., 2021). Among the “3 medicines”, the composition of Jinhua Qinggan Granule and Lianhua Qingwen Granule/Capsule is modified from Yinqiao Powder and Maxing Ganshi Decoction; the composition of “3 formulations” also includes Maxing Ganshi Decoction. Yinqiao Powder and Maxing Ganshi Decoction are two well-known classical Chinese medicine prescriptions, and their efficacy in the treatment of lung diseases have been proven by many studies (Mei et al., 2014; Wang et al., 2020a; Li, 2020). Yinqiao Powder (from *Systematized Identification of Warm Diseases* 溫病條辨) is composed of ten botanical drugs, i.e., *Lonicerae Japonicae Flos* (Jin-yin-hua 金銀花), *Forsythiae Fructus* (Lian-qiao 連翹), *Platycodi Radix* (Jie-geng 桔梗), *Menthae Haplocalycis Herba* (Bo-he 薄荷), *Lophatheri Herba* (Dan-zhu-ye 淡竹葉), *Schizonepetae Spica* (Jing-jie-sui 荊芥穗), *Sojae Semen Praeparatum* (Dan-dou-chi 淡豆豉), *Arctii Fructus* (Niu-bang-zi 牛蒡子), *Phragmitis Rhizoma* (Lu-gen 蘆根) and *Glycyrrhizae Radix et Rhizoma* (Gan-cao 甘草), which can release the exterior with pungent-cool (辛涼透表) and clear heat-toxin (清熱解毒) (Wang, 2013). On the other hand, Maxing Ganshi Decoction (from *Treatise on Cold Damage and Miscellaneous Diseases* 傷寒雜病論) is a combination of three botanical drugs and one mineral drug, including *Ephedrae Herba* (Ma-huang 麻黃), *Armeniacae Semen Amarum* (Ku-xing-ren 苦杏仁), *Gypsum Fibrosum* (Shi-gao 石膏) and *Glycyrrhizae Radix et Rhizoma* (Gan-cao 甘草) which can diffuse the lung and discharge heat (宣肺泄熱) and suppress cough and calm panting (止咳平喘) (Zhang, 2013). A prospective randomized controlled trial showed that the combination of Yinqiao Powder and Maxing Ganshi Decoction were effective in reducing the time of fever resolution caused by influenza A virus H1N1 (Wang et al., 2011).

There has been much focus on CM for the treatment of influenza viruses and several correlational studies have been conducted on this topic. According to the experimental data, CM has the ability to inhibit virus replication by destroying the virus directly, interfering with virus adsorption and protecting cells from virus infection (Lu et al., 2008). In 2003, there was an outbreak of the Severe Acute Respiratory Syndrome (SARS). At that time, 40%–60% patients in the mainland received CM treatment alongside standard medical care, and good results were obtained (Leung, 2007). A research showed that CM could enhance immunoregulatory function and prevent infection effectively including SARS (Poon et al., 2006). According to CM theory, COVID-19 belongs to “pestilence (疫病)” (Zhang et al., 2020; Zhang, 2022; Zhi et al., 2022). The main clinical manifestation is fever, dry cough, fatigue and upper respiratory infection symptoms, which can be treated by the combination of Yinqiao Powder and Maxing Ganshi Decoction (National Health Commission of the People’s Republic of China, 2022). In fact, since the outbreak of the fifth wave of COVID-19 in Hong Kong, these two formulae have often been used with modifications by CM practitioners in the treatment of acute phase of COVID-19. The “Special Chinese Medicine Programme for COVID-19 In-patients” launched by the Hong Kong Hospital Authority clearly specifies the use of modified “Yinqiao Powder” and “Qingfei Paidu Decoction”, which include Maxing Ganshi Decoction, for the treatment of patients with specified types of CM patterns (證型) (Hospital Authority, 2022). However, there is still a lack of high-quality clinical evidence on the effectiveness and safety of the combination of Yinqiao Powder and Maxing Ganshi Decoction in the treatment of COVID-19. Therefore, this study will use the combination of these two formulae as the intervention for the treatment of COVID-19.

During the COVID-19 pandemic, telemedicine is one of the most effective ways of dealing with and controlling the COVID-19 in many countries, in order to ensure the continuity of care for patients with chronic diseases and timely supportive care for COVID-19 patients (Colbert et al., 2020). Patients are able to have the medical consultation by video-conference and receive the medication by delivery service, which can reduce the risk of transmission by minimizing direct contact. Therefore, telemedicine services have also been provided by the Hospital Authority (HA) and some private hospitals or clinics in Hong Kong (Whitehead, 2020). The fifth wave of the COVID-19 epidemic occurred in Hong Kong in 2022 has caused significant burden to the healthcare system. The Integrative Medical Centre of Hong Kong Institute of Integrative Medicine launched in the same year the “Free Telemedicine Consultation Service for COVID-19 Patients”, a programme providing CM treatment for COVID-19 patients through telemedicine. We plan to initiate a clinical study to test the effectiveness and safety of combined Yinqiao Powder and Maxing Ganshi Decoction for the treatment of COVID-19 patients.

## Study aim

The study aims to 1) evaluate the effectiveness and safety of Yinqiao Powder-Maxing Ganshi Decoction in the treatment of the major symptoms of mild and moderate COVID-19 patients by telemedicine; 2) provide a scientific rationale for the use of Yinqiao Powder-Maxing Ganshi Decoction in the treatment of COVID-19; 3) raise the awareness of Chinese medicine among COVID-19 patients and the wider community.

## Hypothesis

The combined Yinqiao Powder-Maxing Ganshi Decoction is superior to placebo in improving the major symptoms of mild and moderate COVID-19.

## Methodology

### Study design

This is a randomized, double-blinded, placebo-controlled trial via telemedicine. Eligible subjects will receive a maximum of 14 days’ treatment with Yinqiao Powder-Maxing Ganshi Decoction (with or without specified additions) or placebo, and will be followed up on days 7, 14 and 35 after receiving medication (day 0).

### Study population

Subjects who are diagnosed with COVID-19 by Rapid Antigen Tests (RATs) or PCR tests based on criteria of the Centre for Health Protection (CHP) of the Department of Health (DH), Hong Kong SAR, China will be recruited via telemedicine from the following clinics/Chinese medicine centres: 1) The CUHK Chinese Medicine Specialty Clinic and Teaching and Research Centre on CUHK campus (CUHK-CMSCTRC); 2) The two Integrative Medical Centres, Hong Kong Institute of Integrative Medicine at Shatin and Wan Chai respectively. Advertisements in the form of posters on the recruiting clinics/centres and internet platforms, such as Facebook, emails, instant messaging applications and websites will be made to facilitate community recruitment. Besides, we will publish articles in local newspapers and magazines as well as organize health promotion talks to augment the subject recruitment process. We will also seek to promote the trial in other suitable mass media if possible and necessary. Potential subjects who meet the eligibility criteria will be recruited.

### Eligibility

Subjects who fulfill all of the inclusion criteria and have none of the exclusion criteria will be recruited.

Potential suitable subjects with COVID-19 will be screened for the following eligibility criteria:

### Inclusion criteria

1. Age from 18 to 65 years;
2. Tested positive for COVID-19 by RATs, Deep Throat Saliva (DTS) or Throat/Nasal swab RT-PCR tests;
3. Patient diagnosed with mild to moderate COVID-19 based on the “Coronavirus disease 2019 (COVID-19) Treatment Guidelines” published by National Institutes of Health, United States;
4. Duration of symptoms ≤72 h (Start from the presence of the major symptoms of COVID-19: Fever: oral temperature >37.2°C, or cough score ≥2, or fatigue score ≥2);
5. At the moment of screening (or within 2 h before screening), body temperature: >37.2°C
6. Diagnosed with pattern of wind and heat invading the exterior (風熱犯表證) and/or pattern of intense exuberance of lung heat (肺熱熾盛證) by a registered Chinese medicine practitioner (CMP) (Diagnostic criteria are the corresponding criteria in the “Diagnosis standards for common syndromes in traditional Chinese medicine (《中醫常見證診斷標準》)” published by “Diagnostic Subcommittee of China Association of Traditional Chinese Medicine (中華中醫藥學會中醫診斷學分會)” in 2008, with ≥20 as the threshold for determination);
7. Explicit declaration of willingness to participate in the study at the time of video-conference screening after reading the electronic informed consent form (written consent form will have to be signed after inclusion).

### Exclusion criteria

• Diagnosed with Asymptomatic or Presymptomatic Infection, Severe Illness or Critical Illness of COVID-19 based on the “Coronavirus disease 2019 (COVID-19) Treatment Guidelines” published by National Institutes of Health, United States;
• Known allergy to the Chinese medicines or other ingredients of the investigational medicinal products (IMP) used in this study;
• Known pregnancy or lactation;
• Known immunocompromised patient (such as malignancy, organ or bone marrow transplant, AIDS or low immune function caused by long-term use of corticosteroids or other immunosuppressants)
• Known obesity (Body Mass Index [BMI] ≥30)
• Heavy smoker (smoking index ≥400 [cigarettes smoked per day × years of tobacco use])
• Known history of cardiovascular and cerebrovascular diseases (including hypertension), chronic lung disease (chronic obstructive pulmonary disease, moderate and severe asthma or interstitial lung disease), diabetes, chronic liver disease (ALT/AST ≥2× the upper limit of normal [ULN], Bilirubin-Total ≥1.5ULN), chronic kidney disease (Creatinine ≥1.5ULN), cancer;
• Known presence or suspicion of acute liver or kidney diseases;
• Known history of dysphagia or any gastrointestinal disorder that affects drug absorption (such as gastroesophageal reflux disease [GERD], chronic diarrhea, inflammatory bowel disease, intestinal tuberculosis, gastrinoma, short bowel syndrome, gastrectomy);
• Suspected or known history of alcohol or substance abuse or mental illness;
• Subjects having participated in other clinical studies in the past 3 months; and
• Any other condition that in the opinion of the investigators could compromise the study.

## Study intervention

### Study medications

Yinqiao Powder-Maxing Ganshi Decoction is the core formula in this clinical study and consists of the following botanical and mineral drugs: Ephedra sinica Stapf/Ephedra intermedia Schrenk and C.A.Mey./Ephedra equisetina Bunge [Ephedraceae; Ephedrae Herba Praeparata cum Melle] (Mi-ma-huang 蜜麻黃) 6 g, Prunus armeniaca var. armeniaca/

Prunus sibirica L./Prunus mandshurica (Maxim.) Koehne/Prunus armeniaca L. [Rosaceae; Armeniacae Semen Amarum] (Ku-xing-ren苦杏仁) 6 g, Glycyrrhiza uralensis Fisch. ex DC./Glycyrrhiza inflata Batalin/Glycyrrhiza glabra L. [Fabaceae; Glycyrrhizae Radix et Rhizoma] (Gan-cao 甘草) 10 g, Gypsum Fibrosum (Shi-gao 石膏) 12 g, *Lonicera japonica* Thunb. [Caprifoliaceae; Lonicerae Japonicae Flos] (Jin-yin-hua 金銀花) 15 g, Forsythia suspensa (Thunb.) Vahl [Oleaceae; Forsythiae Fructus] (Lian-qiao 連翹) 15 g, Platycodon grandiflorus (Jacq.) A. DC. [Campanulaceae; Platycodonis Radix] (Jie-geng 桔梗) 10 g, Mentha canadensis L. [Lamiaceae; Menthae Haplocalycis Herba] (Bo-he 薄荷) 6 g, Lophatherum gracile Brongn. [Poaceae; Lophatheri Herba] (Dan-zhu-ye 淡竹葉) 15 g, Nepeta tenuifolia Benth. [Lamiaceae; Schizonepetae Spica] (Jing-jie-sui荊芥穗) 12g, Glycine max L.) Merr. [Fabaceae; Sojae Semen Praeparatum] (Dan-dou-chi 淡豆豉) 15 g, Arctium lappa L. [Asteraceae; Arctii Fructus] (Niu-bang-zi 牛蒡子) 15 g, Phragmites australis subsp. australis [Poaceae; Phragmitis Rhizoma] (Lu-gen蘆根) 30 g. At the same time, the following additions will be made according to the symptoms and pathogenesis of the patient at inclusion, in order to reflect the principle of pattern identification of Chinese medicine theory:

i. Sore throat (heat accumulation in the lung and stomach熱蘊肺胃):
  • Isatis tinctoria subsp. tinctoria [Brassicaceae; Isatidis Radix] (Ban-lan-gen 板藍根) 15 g,
  • Ilex asprella Champ. ex Benth. [Aquifoliaceae; Ilicis Asprellae Radix] (Gang-mei-gen崗梅根) 15 g, Lasiosphaera Calvatia (Ma-bo 馬勃) 5 g;
ii. Cough and asthma with yellow sputum or less sputum (phlegm and heat obstructing the lung痰熱阻肺): Fritillaria thunbergii Miq. [Liliaceae; Fritillariae Thunbergii Bulbus] (Zhe-bei-mu 浙貝母) 10 g, Trichosanthes kirilowii Maxim./Trichosanthes rosthornii Harms [Cucurbitaceae; Trichosanthis Pericarpium] (Gua-lou-pi 瓜蔞皮) 10 g;
iii. Nausea and vomit, torpid intake (納呆) and sloppy stool (便溏) (dampness impacting the middle energizer濕阻中焦): Pogostemon cablin (Blanco) Benth. [Lamiaceae; Pogostemonis Herba] (Guang-huo-xiang 廣藿香) 10 g, *Poria* (Fu-ling 茯苓) 10 g, Atractylodes macrocephala Koidz. [Asteraceae; Atractylodis Macrocephalae Rhizoma] (Bai-shu白术) 10 g;
iv. Insomnia (Heat harassing the heart spirit 熱擾心神): Ziziphus jujuba Mill. [Rhamnaceae; Ziziphi Spinosae Semen] (Suan-zao-ren酸棗仁) 10 g,
v. Reynoutria multiflora (Thunb.) Moldenke [Polygonaceae, Polygoni Multiflori Caulis] (Shou-wu-teng首烏藤) 10 g, Margaritifera Concha (Zhen-zhu-mu 珍珠母) 20 g.

Subjects in the treatment group will receive CM granules in dosages equivalent to the decocting piece dosages shown above according to the IMP manufacturer’s illustration (estimated granule weight per dose: 33.4–59.7 g), and will be dissolved in hot water and administered while it is warm. Participants will take 1 dose a day, and the medication will be divided into two portions, with one in the morning and the other in the evening after meal. The time of treatment and medication is 14 days at maximum or until patients get negative RAT results on two successive days and the major symptoms subside (define as oral temperature ≤37.2°C, the score of cough and fatigue ≤1). The actual duration is determined by the patient according to their own condition, and they can also seek advice from the research team or registered CMPs though the direct phone line if necessary.

The CM granules are planned to be a mixture of single granules produced by PuraPharm, a commercial company in Hong Kong. Most of these granules are made from botanical drugs used in CM, with some made from fungi (*Lasiosphaera Calvatia, Poria*), animal (*Margaritifera Concha*), and mineral (*Gypsum Fibrosum*). They are turned into granules by using modern methods that mimic the old way of making CM decoction. PuraPharm is the main provider of these granules to Hong Kong’s hospitals and health institutions. The botanical, animal and mineral drugs meet the standards of Chinese Pharmacopoeia if they are included. This company follows the GMP standards of the PRC, Australia and the US for quality control and production. It also has an ISO 17025 certified laboratory to check the quality.

The placebo was made from Sorbitol, Maltodextrin, Corn Starch, Soluble Fiber, Black Bean Extract, Abelmoschus esculentus Extract, Green Tea Extract, Caramel Color Powder, Citric Acid and Ginseng Flavo, whose appearance, smell and taste was similar to the CM granules. Subjects will be instructed to take them in the same way as in the treatment group. Each study subject will be given relevant information on the use and storage of IMPs. The granules of both treatment and placebo group will be produced by a manufacturer with a GMP certificate.

According to Chinese Medicine Council of Hong Kong’s official website (Chinese Medicine Council of Hong Kong, 2023) “If the single Chinese medicine granules are only supplied to Chinese medicine practitioners to dispense a prescription, instead of dispensing the herbs, they do not fulfill the definition of pCm (*proprietary Chinese medicine*) and thus are not required to apply for pCm registration”. For this reason, the concentrated Chinese medicine granules used in this study are not pCms, and therefore no Certificate for Clinical Trial and Medicinal Test (CTC) is needed for this clinical study.

### Possible risks and discomfort

The *Lonicerae Japonicae Flos* in Yinqiao Powder contains saponins, which generally had hemolytic effect (Pan et al., 2005; Zhang et al., 2014). However, the saponins contained in *Lonicerae Japonicae Flos* have no or weak hemolytic effect and the potential risk of haemolysis in clinical application is low (Liang et al., 2018). On the other hand, some studies reported that the *Ephedrae Herba* of Maxing Ganshi Decoction contains ephedrine, which consumed in excess can cause a reduction in elimination of adrenaline, resulting in excitation of the sympathetic and central nervous systems (Ou and Wang, 2005; Huang et al., 2017). However, there is no known report on the adverse drug reaction due to *Ephedrae Herba* in the literature related to Maxing Ganshi Decoction (Lin, 2022; Wu et al., 2022). In addition, no severe adverse reaction has been reported in the current literature even though Yinqiao Powder and Maxing Ganshi Decoction are commonly used in the treatment of COVID-19 (Wang et al., 2020c).

### Study visits

#### Screening

Subjects who express interest in our study will be invited to do the screening by video-conference. The electronic informed consent form will be sent to them before the screening. The information about the study will be explained to the participants by our research staff. The eligibility assessment will only be conducted when subjects explicitly indicate that they have read the electronic informed consent form and volunteered to participate in the study at video screening. The written consent form has to be signed after inclusion at the appropriate time. To address exclusion criterion 7 on chronic liver and kidney disease, participants will be screened for chronic liver or kidney disease by asking about their past medical history. If patients’ answer is “Yes”, they will be excluded immediately; if “No”, then we will ask if any blood tests have been done in the past 6 months. If they answer “No”, the participants will be assumed to have no known liver or renal impairment and screening can continue. However, if the answer is “Yes”, then the participants who have blood test reports will be determined based on the results; For those without reports, they will be asked if any medical professional has indicated any abnormalities in liver or kidney function, and those with “Yes” will be excluded, while those with “No” will be screened on the assumption that there is no known liver or kidney impairment. In addition, if patients with symptoms such as jaundice, dark urine and oliguria, which are highly suspicious of liver and kidney impairment, will be excluded and advised to seek medical advice.

#### Baseline randomization (day −1 or 0)

Eligible subjects will be assessed by our registered CMPs via telemedicine in this trial, during which medical history and concomitant medication will be recorded. After stratification, subjects will be randomly assigned in a 1:1 ratio to receive up to 14 days of either modified Yinqiao Powder-Maxing Ganshi Decoction (about 33.4–59.7 g each day) or placebo granules (about 33.4–59.7 g each day). Specifically, the stratified randomisation is based on age (A: “18–49 years old” and B: “50–65 years old”) and the number of vaccination (a: “received 3 or more doses - fulfilling government vaccination requirement” and b: “received 2 doses or less of vaccine - yet to be fully vaccinated”). Subjects will be divided into two groups by age: one group is “18–49 years old” and the other one is “50–65 years old”; then they will be further divided into two groups within the different age group by the numbers of vaccination: one group is “received 3 or more doses of vaccine, and the another is “received 2 doses or less of vaccine”. Four subgroups will be created after stratification and subjects in each subgroup will be randomly assigned to either the treatment or placebo group. The distribution of age and number of vaccinations between the two groups will be roughly comparable after stratified randomisation. The IMP will be prescribed by Registered CMPs and will be sent to patients by delivery service. The current Western medicine treatment can continue during the study period.

#### Follow-up assessment (Days 7, 14) and end-of-study assessment (Day 35)

Subjects will have video consultation on day 7 (±1 day) and day 14 (±1 day) and post treatment follow up at day 35 (±1 day) (Table 1). Subjects are required to measure their body temperature and oxygen saturation twice daily; conduct RATs, clinical symptom score measurement and tongue image capture once daily during the day 0–14 (or until the body temperature returns to normal for 24 h/the day the RAT is negative for 24 consecutive hours, whichever is later). Subjects also need to score their symptoms and capture their own tongue image at day 35. All subjects are required to keep a daily record on their body temperature, oxygen saturation, result of RATs, symptom scores, IMP compliance and adverse events. A direct telephone line will be provided so that subjects can report any adverse event during office hours. The subjects will be recommended to attend Emergency Department at the nearest hospital beyond office hours if deemed necessary. In order to ensure compliance, supermarket shopping vouchers worth 300 Hong Kong dollars will be provided to all subjects by registered mail upon completion of the study. Compliance with medication will be assessed by asking subjects about their remaining medication on the remote assessment (days 7, 14 and 35) and by patient diaries completed by the subjects which will be sent back to the research team after the day 35 assessment.

**TABLE 1:** Study schedule.

Items	Screening	Treatment period			End of study visit
	−1 to 0 days	Day 0	Day 7 (±1 days)	Day 14 (±1 days)	Day 35 (±1 days)
Explicit consent on virtual screening	X				
Eligibility	X				
Medical consultation and assessment	X		X	X	X
Medical history	X				
Concomitant medication	X		X	X	X
AE/SAE assessment			X	X	X
Prescription and dispensing of IMP	X				
Medication		X (Max: 14 days)			
Body temperature and oxygen saturation measurement, RAT and tongue image taken		X (14 days or until recovery)			X
Subjects’ daily record for study		X			

### Study schedule

Please see Table 1.

### Study outcomes

#### Assessment

1. The time between starting medication and body temperature returning to normal and the recurrence rate of fever after momentary recovery. Each subject will be issued with the same type of oral thermometer for measurement at 9 a.m. (±2 h) and 9 p.m. (±2 h) daily, with at least 12 h between each measurement. Normal oral temperature is defined as equal to or below 37.2°C. Temperature recovery time is defined as the total number of days between medication starting date and the date of “normal oral temperature for 24 h without antipyretic” (both days inclusive). Subjects are required to measure the temperature until day 14 or on the day when the temperature has remained normal for 24 h, whichever is later.
2. The negative rate of RATs at day 14 and negative conversion time. Each subject will be issued with the same type of RAT kits which can be used for at least 14 days for testing at 9 a.m. (±2 h) each day, with at least 24 h between each test. Negative conversion time is defined as the total number of days between the medication starting date and the date of securing two successive negative RAT results (both days inclusive). Subjects were required to undergo testing until day 14 or the day when two successive negative RAT results at least 24 h apart are obtained, whichever is later.
3. The remission rate at day 14 and remission time of major symptoms.
4. The disappearance rates at day 14 and disappearance time of symptoms. Subjects are required to record the status of their symptoms including major clinical symptoms daily at 9 p.m. (±2 h). Except for fever which is marked as ‘No’ or ‘Yes’ according to whether the oral temperature exceeds 37.2°C, other symptoms will be graded on a scale of 0–4 from “None”, “Mild”, “Moderate”, “Severe” and “Very Severe” depending on the actual feelings of the subject (“None” means the symptom is not present, “Mild” means the symptom is present but does not interfere with daily activities; “Moderate” means the symptom is present and slightly interferes with daily activities; “Severe” means the symptom is present and most daily activities cannot be done; “Very Severe” means the symptom is present and interferes with all daily activities). Major clinical symptoms include fever, cough and fatigue. Remission is defined as oral temperature ≤37.2°C, cough and fatigue score ≤1; disappearance is defined as the symptom being present at day 0 (score >0) but the symptom score is 0 at the date of recording.
5. All-cause mortality at the 21st day after stopping the medication.
6. Incidence of the progression to severe or critical illness during the trial. The all-cause mortality at the 21st day after stopping the medication and the incidence of the progression to severe or critical illness during the trial will be confirmed by communication with the subjects and/or other related person(s) or checking their medical record in the Hospital Authority. The definitions of severe and critical illness are based on the related definitions of “Coronavirus disease 2019 (COVID-19) Treatment Guidelines” published by National Institutes of Health (severe illness: oxygen saturation in the indoor environment of sea level <94%, oxygenation index [PaO2/FiO2] < 300 mmHg, respiratory frequency >30 breaths/min, lung infiltrates >50%; critical illness: respiratory failure, septic shock, and/or multiorgan dysfunction or failure).
7. Utilization rates of antipyretic, Paxlovid and molnupiravir within 14 days and the total dosage(s) of the drug(s) used. In this study, subjects are allowed to take western medicines prescribed for the treatment of COVID-19 under the current guidelines of the Hong Kong Hospital Authority with at least 2 h’ gap between Chinese and western medicines. If oral temperature >39.0°C and continues over 4 h; or the oral temperature >39.5°C, the patient is advised to take antipyretic (paracetamol is recommended as a priority) for acute treatment. Patients need to record the amount and time of the drug(s) used.
8. Oxygen saturation. For safety reasons, each subject will be issued with the same type of pulse oximeter for measurement at 9 a.m. (±2 h) and 9 p.m. (±2 h) daily, or whenever the subject decides it is necessary. If the reading is less than 94%, the patient is considered to have severe illness and has reached the end point of the study and is recommended to seek medical attention immediately. Subjects are required to measure their oxygen saturation at rest until day 14 or the time of body temperature returning to normal (continuing for 24 h) or the negative conversion time (2 successive negative RAT results), whichever is later.

### Primary outcome

The time between starting medication and body temperature returning to normal (oral temperature 37.2°C and maintain 24 hours).

### Secondary outcomes

1. The recurrence rate of fever after momentary recovery: the incidence of fever recurrence (oral temperature > 37.2°C) within 24 hours after the patient’s temperature has normalized after treatment;
2. The negative rate of RATs at day 14 and the negative conversion time (two successive negative RATs result with at least 24 hours interval);
3. The remission rate of major symptoms at day 14 (oral temperature ≤ 37.2°C, score of cough and fatigue ≤ 1 (“None”, “Mild”, “Moderate”, “Severe” and “Very severe” were marked as 0 to 4 450 respectively), and continues 24 hours);
4. Time of major symptoms remission: time from the start of treatment to symptoms remission (definition of symptoms remission: oral temperature ≤ 37.2°C, cough and fatigue score ≤ 1 and lasting at least 24 hours);
5. The disappearance rates at day 14 and disappearance time of symptoms: the disappearance rates and time of individual symptoms of mild and moderate COVID-19 within 14 days (time defined as the period between starting medication and symptom disappearance);
6. The rate of patients whose oxygen saturation had gone below< 94% after intake of drugIMP, measured at day 14;
7. Incidence of the progression to severe or critical illness during the trial;
8. All-cause mortality at the 21st day after stopping the medication;
9. Utilization rates of antipyretic, Paxlovid and molnupiravir within 14 days and the total dosage(s) of the drug(s) used.

### Safety index:

1. Oxygen saturation;

2. Rate of adverse events

### Sample size and statistical methods

#### Sample size estimation

The primary outcome of the study is the time between starting medication and body temperature returning to normal, i.e., the time to fever resolution after the first treatment. Based on previous studies, we assumed the standard deviation (SD) is 0.7, and the time to fever resolution of half a day (0.5 days) would have clinical significance (Qiu et al., 2020; Chen et al., 2021). Therefore, to detect such a difference between groups, 38 in each group will be needed with a power of 80% and a 5% 2-tail significance level. To allow for possible dropouts during the study period, we inflate this value by 20%, hence a total of 96 patients will be needed for the proposed study and 48 in each group (12 in each sub-group).

#### Randomization and blinding

A random number table will be generated with a computerized random number generator program. The random allocations will be put into opaque envelopes with sequential study numbers by an independent researcher. Two sets of the envelope will be prepared, one set will be for randomization at the site and another set stored in the investigator’s office for emergency unblinding. Each subject will be assigned with a sequential study number and the corresponding IMP will be prepared according to the allocation by a Chinese medicine pharmacist. In the study, the registered CMP investigators, study subjects and outcome assessors will be kept blind of the allocated intervention.

#### Statistical analysis

Basic demographic data (gender, age, *etc.*) will be descriptively summarized. Continuous variables are presented as mean ± standard deviation (SD) and categorical variables are presented as ratios. Intra group comparisons between baseline and each visit will be conducted by using paired *t*-test (or Wilcoxon signed rank test). Linear mixed model will be used to compare the outcomes between groups. All statistical tests will be two-sided, and *p* < 0.05 is considered significant difference. The statistical analysis will be done by an independent statistician and all analysis will be conducted by SPSS. If there is a significant difference in the use of western medicines between the treatment and control groups, it will be considered and processed as a covariance in the statistical analysis.

In this study, the subject will be deemed as “per protocol” if they have taken the maximum dose or if they stop until getting negative RAT results on two successive days and the major symptoms subside (defined as oral temperature ≤37.2°C, the score of cough and fatigue ≤1) with ≥85% intake compliance. The intake compliance is calculated by dividing the no. of packs of drugs ingested by either:

a) the number of packs of core formula the subject should take daily (daily dose) x the number of days from the day of starting drug intake to the day the drug is stopped according to the criteria (e.g., if the subject should take 4 packs of core formula a day, starts taking drug on 1 January, stops at 8 January according to criteria, and has taken 30 packs, the compliance will be (30/(4 × 8)) x 100%) = 93.75%, with the subject deemed as “per protocol”; or
b) daily dose x 14 for all other cases (e.g., if the subject should take 4 packs of core formula a day, starts taking drug in 1 January and stops at 3 January because of adverse events, and has taken 12 packs, the compliance will be (12/(4 × 14)) x 100%) = 21.43%, with subject deemed as not “per protocol”.

#### Adverse events

An adverse event is any undesirable medical event occurring in the subject within the trial period, whether it is related to the study intervention. Although there is no documented evidence indicating any adverse reactions obviously associated with Yinqiao Powder-Maxing Ganshi Decoction, all adverse events during the study will be monitored and recorded according to CTCAE 5.0. The treatment will be suspended when any severe adverse event occurs to ensure subject safety.

#### Subject withdrawal

Subjects can withdraw at any time without having to give any reason. However, our research staff will endeavor to identify the reason(s) and these subjects will be encouraged to attend the follow-up at day 35 or an unscheduled follow-up for assessment to the benefit of subjects.

#### Ethics consideration

Ethical approval will be sought from The Joint Chinese University of Hong Kong–New Territories East Cluster Clinical Research Ethics Committee before the initiation of this study. Participants will be informed of the measures to ensure the confidentiality of all information, and data anonymity will be maintained. Subjects are required to state categorically that they have read the electronic informed consent and participated voluntarily in the study during the video screening before joining the study. The written consent forms will be signed in due course after they have been recruited. All information will be encrypted and only the involved investigators can have access. Password is required to access the data. Participants are free to withdraw at any time without giving a reason or punishment. The personal data of the subjects will only be kept for 7 years and will be destroyed afterwards.

## Data handling and dissemination of information

To protect patient privacy, all research documents will be locked in cabinets where the clinics keep the patients’ consultation notes. All collected information will be input into a computer with restricted access to the research team only. Only de-identified data will be collected and removed from the premises. The Clinical Research Management Office at The Chinese University of Hong Kong or an external auditor will perform auditing or inspection to determine whether research activities are conducted according to the protocol, Good Clinical Practice (GCP) and guidelines of the International Conference on Harmonization (ICH).

### Clinical trial insurance

Clinical trial insurance will be purchased according to the University’s policy.

## Discussion

Although COVID-19 situation is now stable and considered as an endemic disease in many places including Hong Kong, it still remains a global health issue and many people are still affected. As at 20 April 2023, there was a rise in the positive detection rate of COVID-19 and the severity and fatalities cases (Centre for Health Protection, 2023; World Health Organization, 2023). Therefore, it is important to explore more effective and safe treatments in order to further minimize the impact of COVID-19 and improve the public health. A review found that there have been many clinical trials for COVID-19 with prescriptions including Yinqiao Powder or Maxing Ganshi Decoction (Kang et al., 2022). However, there is a lack of studies on the use of Yinqiao Poweder and Maxing Ganshi Decoction alone in treating COVID. This randomized double-blind placebo clinical study is designed to evaluate the efficacy and safety of Yinqiao Powder-Maxing Ganshi Decoction in alleviating the symptoms of COVID-19.

The treatment of COVID-19 in CM does not only focus on “combating the virus”, but also needs to be determined by the patients’ physical condition, symptoms, syndrome, the season and the geographical location, which is called “treatment individualized to patient, season and locality” (三因制宜).

In Maxing Ganshi Decoction, *Ephedrae Herba Praeparata cum Melle* and *Armeniacae Semen Amarum* can help relieve breathing problems, chest congestion and coughing and seem to be effective in treating shortness of breath and sputum after COVID-19 infection. Yinqiao Powder, on the other hand, is effective in clearing heat and removing toxin (清熱解毒), and it has anti-viral and anti-inflammatory effects for many respiratory and infectious diseases. A study proved that the *Forsythiae Fructus* has anti-influenza effect (Su et al., 2010).

In this study, subjects are required to keep a record daily for their vital signs such as oral temperature and oxygen saturation, RAT results, tongue imaging and symptoms score while taking the study medicine as well as regular video follow up (Day 7, day 14 and Day 35), which can clearly show the patient’s physical condition and help in subsequent assessment and statistics.

As telemedicine is the primary consultation method for this study, subjects will not visit the clinic throughout the study period and all communication with subjects will be via internet and delivery service. There is a risk of privacy breaches if documents are lost. Therefore, the postal documents in this study will only be marked with a code and abbreviation, and personal details will be filled in for the identification purposes after they are returned.

Since the use of other medications for COVID-19 is not prohibited in this study, the results of the study may be influenced by other medications taken by the subjects rather than this study medication. For this reason, the amount and using time of other medication must be recorded and become an important outcome for statistics, so that we could have a more accurate result of the effect of Yinqiao Powder-Maxing Ganshi Decoction.

We are aware that shedding of dead virus might still occur after patients have recovered from all major symptoms. However, we still think including the negative rate of RATs at day 14 and negative conversion time as secondary outcomes are valuable, for according to one study which investigated 14 different brands of RATs, RATs have very high specificity (100%) for all Ct values, and high sensitivity (88.18%) when Ct value is at or below 30, a threshold above which no virus can be cultured according to another study (Young et al., 2020; Routsias et al., 2021). Their sensitivity lowers to 37.5% when Ct values are from 31 to 35 (Routsias et al., 2021). This suggests RAT positivity is highly correlated with infectiousness, in sharp contrast to PCR tests, which detect non-infectious viral materials for an extended period post infection (Widders et al., 2020). Such properties of RATs have lead a New Zealand expert to deem RATs as the ideal option for “test-to-release” schemes to be adopted by governments for infection control (Science Media Centre, 2022). In fact, the Hong Kong and United Kingdom governments have adopted such schemes during the pandemic (GOV.UK, 2022; Information Services Department, 2022).

In this study, we will distribute RAT kits to subjects for their use and no PCR test will be conducted in the study period. Therefore, we think the rate of false positive by detecting dead virus can be minimized, and these outcomes will be of interest to policymakers and public health researchers.

As far as we are aware of, this study is the first randomized, double-blind, placebo-controlled study on the efficacy and safety of Yinqiao Powder-Maxing Ganshi Decoction on the acute phase of COVID-19. It is anticipated that this study will raise awareness among the scientific community of the potential of Chinese medicine in treating COVID-19, and establish it as a potential alternative to the current anti-viral treatments. This is especially important to developing countries with socioeconomic profiles different from western developed countries.

In addition, individuals with a higher risk of serious adverse reactions or developing severe or critical illnesses will be excluded from this study in order to ensure the subjects’ safety, minimize potential adverse effects and enhance the feasibility of data collection and follow-up visits. While these exclusions may limit the generalizability of our findings, they were necessary to maintain participant safety and data validity. Nevertheless, future studies can be designed to specifically address the safety concerns in these excluded populations as they are most likely to benefit from the treatment.
